# Comparison of glycyrrhizin content in 25 major kinds of Kampo extracts containing Glycyrrhizae Radix used clinically in Japan

**DOI:** 10.1007/s11418-017-1101-x

**Published:** 2017-06-12

**Authors:** Mitsuhiko Nose, Momoka Tada, Rika Kojima, Kumiko Nagata, Shinsuke Hisaka, Sayaka Masada, Masato Homma, Takashi Hakamatsuka

**Affiliations:** 1 Department of Pharmacognosy, Faculty of Pharmacy, Meijo University, 150 Yagotoyama, Tempaku-ku, Nagoya, Aichi 468-8503 Japan; 2 Division of Pharmacognosy, Phytochemistry and Narcotics, National Institute of Health Sciences, 1-18-1 Kamiyouga, Setagaya-ku, Tokyo, 158-8501 Japan; 3 Department of Pharmaceutical Sciences, Faculty of Medicine, University of Tsukuba, 1-1-1 Tenno-dai, Tsukuba, Ibaraki 305-8575 Japan

**Keywords:** Glycyrrhizae Radix, Glycyrrhizin (GL), Kampo extracts, HPLC, pH, Pseudoaldosteronism

## Abstract

Glycyrrhizae Radix is the most frequently used crude drug in Japan and is prescribed in Kampo medicine for the treatment of a wide range of diseases. The major active ingredient of Glycyrrhizae Radix, glycyrrhizin (GL), has been shown to possess various pharmacological actions, but is also known to cause adverse effects such as pseudoaldosteronism. To avoid the adverse effects of GL, precautions have been indicated on the package inserts of Glycyrrhizae Radix-containing formulas depending on the amount of Glycyrrhizae Radix they contain. However, it remains unknown whether the extraction efficiency of GL from Glycyrrhizae Radix is constant throughout the different combinations of crude drugs in Glycyrrhizae Radix-containing formulas. To confirm the basis of the safety regulation, in this study we comprehensively determined the GL content of 25 major kinds of Kampo extracts compounding Glycyrrhizae Radix. We found that the GL content per daily dosage in all Kampo extracts are generally proportional to the compounding amount of Glycyrrhizae Radix, except in the case of shoseiryuto (Sho-seiryu-To). We also found that Schisandrae Fructus in Sho-seiryu-To decoction caused a lowered pH condition and drastically decreased the extraction efficacy of GL from Glycyrrhizae Radix. Moreover, we were able to confirm that the extraction efficiency of GL from Glycyrrhizae Radix is dependent on the pH value of the extraction solvent. The extraction efficiency of GL in the 25 kinds of Kampo extracts was not constant but it correlates significantly with the pH value of the decoction. Furthermore, the GL contents are well correlated with pseudoaldosteronism incidence data obtained from the Japanese Adverse Drug Event Report (JADER) database on the 25 kinds of Kampo extracts. This suggests that the GL content is a better index to consider to avoid the adverse effects of Glycyrrhizae Radix-containing Kampo formulas.

**Electronic supplementary material:**

The online version of this article (doi:10.1007/s11418-017-1101-x) contains supplementary material, which is available to authorized users.

## Introduction

Glycyrrhizae Radix is the most frequently used crude drug in Japan and is defined in the Japanese Pharmacopoeia as the root and stolon of *Glycyrrhiza uralensis* Fischer or *Glycyrrhiza glabra* Linne. The root of *Glycyrrhiza* plants has also been used as natural food additives for a long time [1]. Glycyrrhizae Radix is prescribed as an active component in multi-drug formulations of Kampo medicine to treat a variety of diseases.

Glycyrrhizin (GL), a major ingredient of Glycyrrhizae Radix, is a triterpenoid saponin [2] and shows various pharmacological actions such as anti-inflammatory [3–5] and anti-allergy effects [6–8]. Purified GL is also used as a medicine for the treatment of chronic hepatitis in Japan [9–11], as well as being used as a sweetener of natural origin [12].

Several investigators have reported that excessive and/or long-term administration of Glycyrrhizae Radix-containing Kampo medicines and crude drug products or GL alone frequently leads to pseudoaldosteronism [13–15], such as peripheral edema [16–18], hypokalemia [19, 20] and hypertension [21, 22]. These adverse effects are thought to result from glycyrrhetinic acid (GA), a major metabolite of GL that inhibits type 2 11β-hydroxysteroid dehydrogenase, leading to an increase in cortisol level instead of cortisone in the kidney. The elevated cortisol stimulates the mineralocorticoid receptor resulting in increased sodium retention and potassium excretion [23, 24]. In order to avoid these adverse effects, the amount of Glycyrrhizae Radix in Kampo prescriptions is seen as an important factor. Different precautions are described on ethical and OTC drug package inserts depending on the amount of Glycyrrhizae Radix they contain, e.g., more than or less than 2.5 g of Glycyrrhizae Radix in compounding Kampo extract formulation. However, it is still unstated whether the same amount of GL in each Kampo prescription is based on the amount of Glycyrrhizae Radix or not.

Therefore, this study aims to determine how the GL content varies in major Kampo prescriptions, and if the extraction efficiency of GL is affected by other constituents and/or crude drugs in compounding Kampo prescriptions.

In the present study, we chose 25 kinds of Kampo formulas containing Glycyrrhizae Radix from the top 20 list of ethical Kampo formulations and the top 30 list of OTC Kampo formulations in Japan (2011). The Kampo extracts were then prepared and their GL contents were determined by high-performance liquid chromatography (HPLC). Moreover, we calculated and compared the extraction efficacy of each Kampo extract. We also evaluated the relationship between the compounding amounts of Glycyrrhizae Radix or the GL content of the 25 kinds of Kampo extracts and the number of reported cases obtained from the Japanese Adverse Drug Event Report (JADER) database to provide recommendations on how to avoid the adverse effects such as pseudoaldosteronism in Kampo formulas compounding Glycyrrhizae Radix.

## Materials and methods

### Materials

Crude drugs for Kampo prescription were purchased from Tsumura & Co. (Ibaraki, Japan), Tochimoto Tenkaido Co., Ltd. (Osaka, Japan) and Daiko Shoyaku Ltd. (Aichi, Japan). All crude drugs used in this study were Japanese Pharmacopoeia 16th edition (JP XVI) grade. Most of them were crude drugs for preparation of Kampo formulations, and Chrysanthemi Flos and Kasseki were distributed for food and in-pharmacy formulation, respectively. The same lot listed in Table 1 was used throughout all experiments. HPLC-grade acetonitrile and other solvents and chemicals were purchased from Wako (Osaka). A Millipore syringe-driven filter unit (Millex-HP, 0.45 μm pore size) was purchased from Merck Millipore, Ltd. (Darmstadt, Germany). The standard GL (purity >94%, HPLC) was kindly provided by Dr. Yukio Ogihara, emeritus professor at Nagoya City University.

**Table 1:** List of crude drugs used in the study

Name of the crude drug	Lot no.	Manufacturers
Glycyrrhizae Radix	C10891	Tsumura & Co.
Angelicae Radix	F26171	Tsumura & Co.
Asiasari Radix	25039481	Tsumura & Co.
Astragali Radix	24008061	Tsumura & Co.
Bupleuri Radix	F18911	Tsumura & Co.
Ginseng Radix	F3297	Tsumura & Co.
Paeoniae Radix	D49651	Tsumura & Co.
Platycodi Radix	AD2681	Tsumura & Co.
Polygalae Radix	AE4891	Tsumura & Co.
Puerariae Radix	H42281	Tsumura & Co.
Rehmanniae Radix	F00661	Tsumura & Co.
Saposhnikoviae Radix	AE2041	Tsumura & Co.
Saussreae Raidx	3F10M	Daiko Shoyaku Ltd.
Scutellariae Radix	AJ3161	Tsumura & Co.
Alismatis Tuber	AA8921	Tsumura & Co.
Ophiopogonis Tuber	F33241	Tsumura & Co.
Pinelliae Tuber	DO8801	Tsumura & Co.
Atractylodis Rhizoma	F29971	Tsumura & Co.
Cimicifugae Rhizoma	AA9131	Tsumura & Co.
Cnidii Rhizoma	23038151	Tsumura & Co.
Copitidis Rhizoma	AD1381	Tsumura & Co.
Rhei Rhizoma	AJ1911	Tsumura & Co.
Sinomeni Caulis et Rhizoma	F47171	Tsumura & Co.
Zingiberis Processum Rhizoma	AD9651	Tsumura & Co.
Zingiberis Rhizoma	AE5571	Tsumura & Co.
Cinnamomi Cortex	D43061	Tsumura & Co.
Lycii Cortex	022012003	Tochimoto Tenkaido Co. Ltd.
Magnoliae Cortex	AD3571	Tsumura & Co.
Moutan Cortex	AJ9201	Tsumura & Co.
Uncariae Uncis cum Ramulus	AE4331	Tsumura & Co.
Citri Unshu Pericarpium	AE2911	Tsumura & Co.
Euodiae Fructus	25036841	Tsumura & Co.
Forsythiae Fructus	AD2891	Tsumura & Co.
Gardeniae Fructus	AJ8671	Tsumura & Co.
Oryzae Fructus	039007005	Tochimoto Tenkaido Co. Ltd.
Schisandrae Fructus	AJ2741	Tsumura & Co.
Zizyphi Fructus	D35531	Tsumura & Co.
Armeniacae Semen	AA8781	Tsumura & Co.
Longan Arillus	022813003	Tochimoto Tenkaido Co. Ltd.
Nelumbis Semen	F24001	Tsumura & Co.
Plantaginis Semen	1I05M	Daiko Shoyaku Ltd.
Zizyphi Semen	AE0271	Tsumura & Co.
Ephedra Herba	F17391	Tsumura & Co.
Menthae Herba	AA890	Tsumura & Co.
Perillae Herba	AJ0511	Tsumura & Co.
Chrysanthemi Flos	0L13	Daiko Shoyaku Ltd.
Schizonepetae Spica	AD6531	Tsumura & Co.
Polyporus	AD0201	Tsumura & Co.
Poria	F19561	Tsumura & Co.
Asini Corii Collas	3F03M	Daiko Shoyaku Ltd.
Ostreae Testa	AE2701	Tsumura & Co.
Fossilia Ossis Mastodi	AJ7681	Tsumura & Co.
Gypsum Fibrosum	C52151	Tsumura & Co.
Kasseki	OK02	Daiko Shoyaku Ltd.
Natrium Sulfuricum	F00615	Tomita Pharmaceutical Co. Ltd.

### Preparation of Kampo extracts compounding Glycyrrhizae Radix and measurement of the pH values of the decoctions

A daily dosage of crude drugs compounded according to each Kampo formulas was decocted with 600 ml ion-exchanged and distilled water using an electric heater (HMJ-1000N; HARIO Co., Ltd., Tokyo, Japan) for 60 min. The decoction was filtered then cooled down to room temperature. The pH value was measured by a pH meter (SevenEasy pH; Mettler Toledo, Switzerland) using electrodes (InLab Expert Pro). Finally, the filtrate was lyophilized to powder. The extract was stored at −20 °C before use. In the case of Shoseiryuto (Sho-seiryu-To), the extracts of crude drugs and Kampo formulas without one crude drug were prepared in the same manner.

### HPLC analysis of GL content in 25 kinds of Kampo extracts and crude drugs

All procedures were based on JPX VI. Briefly, 50 mg of each powdered Kampo extract was accurately weighed and dissolved with water up to a volume of 50 ml. The mixture was filtered with Millex-HP and then subjected to HPLC analysis. The Shimadzu LC-10Avp HPLC series with UV–VIS detector and C-R8A (Kyoto, Japan) were used for data acquisition and integration. Separations were carried out in an Inertsil-ODS3 (5 μm, 4.6 mm I.D. × 150 mm, GL Science, Tokyo) with 2% acetic acid-acetonitrile (60:40) as eluent. The detection wavelength was 254 nm, the flow rate was 1.0 ml/min, and column temperature was 40 °C. The determination of GL content was carried out by absolute calibration curve method.

### Effect of pH on the extraction efficiency of GL from Glycyrrhizae Radix

With reference to JP XVI, powdered crude drugs were accurately weighed and extracted with 100 mM citric buffer (pH 3.5, 4.0), 100 mM acetate buffer (pH 4.5) or 100 mM phosphate buffer (pH 2.1, 3.0, 5.0, 6.0, 6.8, 8.0) instead of diluted ethanol. Then, the GL content was determined by HPLC.

### Statistical analysis

The correlation analyses (i) between the extraction efficiency and the pH value of the decoction, and (ii) the compounding amounts of Glycyrrhizae Radix or the GL contents and the number of reported cases concerning pseudoaldosteronism, were performed using Pearson’s correlation.

## Results and discussion

### GL content in crude drugs and Glycyrrhizae Radix as a material

First, the GL content in Glycyrrhizae Radix used in this study was determined by HPLC, based on the quantitative method described in JP XVI. It was found that the lot used in this study contained 54.5 ± 2.5 mg of GL per 1 g of dried Glycyrrhizae Radix.

**Fig. 1:**
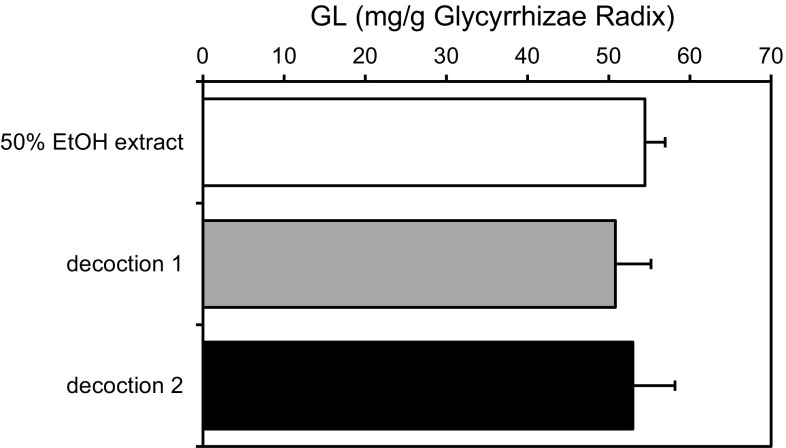
Determination of glycyrrhizin (GL) content in Glycyrrhizae Radix used in this study. The crude drug (Lot No. C10891, Tsumura & Co.) was extracted with 50% ethanol solution as described in JPX VI and the GL content was determined by high-performance liquid chromatography. Decoction 1 was prepared with cut crude drug and decoction 2 was prepared with powdered crude drug. Each *column* represents the mean ± SEM of three samples

It has generally been believed that a higher specific surface area meant higher extraction efficiency in preparing plant extracts to obtain the target compounds using appropriate solvent for a particular material, such as crude drugs. Thus, considering this principle we compared the extraction efficiency of GL in decoction using cut crude drug and powdered crude drug of Glycyrrhizae Radix. Each 2 g of cut crude drug or powdered crude drug prepared from the same lot of Glycyrrhizae Radix was decocted with 600 ml of water for 60 min and the extract was then lyophilized to powder. The yield of the extract and GL content were calculated and compared. We observed that the weight of the extracts was almost the same (average 0.80 g from cut crude drug and average 0.74 g from powdered crude drug) and the GL content was also almost equal, as shown in Fig. 1. These results also revealed that GL can be quantitatively extractable in this condition. The reason why the extraction efficiency was not affected by the size and granularity of the crude drug could be due to the higher water solubility of GL. In any case, we have shown that we do not have to be apprehensive about the difference in extraction efficiency of GL with regard to the size and granularity of the crude drug, Glycyrrhizae Radix, when we prepare and determine the GL content in each Kampo medicine compounding Glycyrrhizae Radix.

### GL content in 25 kinds of Kampo extracts compounding Glycyrrhizae Radix

We chose 25 kinds of Kampo formulas from the top 20 ethical Kampo formulations and top 30 OTC Kampo formulations in Japan (2011) for the determination of GL content in this study (Table 2). In particular, shakuyakukanzoto (Shakuyaku-kanzo-To) shoseiryuto (Sho-seiryu-To) and hangesyashinto (Hange-shashin-To) were selected as formulations compounding 4, 3.0, and 2.5 g of Glycyrrhizae Radix. In addition, gorinsan (Gorin-San), unkeito (Unkei-To), bakumondoto (Bakumondo-To), boiogito (Boi-ogi-To), bofutsushosan (Bofu-tsusho-San), saireito (Sairei-To), shosaikoto (Sho-saiko-To), saibokuto (Saiboku-To), kakkonto (Kakkon-To) and keishikaryukotsuboreito (Keishi-ka-ryukotsu-borei-To) were selected as formulations compounding 2.0 g of Glycyrrhizae Radix, while maoto (Mao-To), saikokeishito (Saiko-keishi-To), seishinrenshiin (Seishin-renshi-In), yokukansankachinpihange (Yokukan-San-ka-chinpi-hange), hochuekikito (Hochu-ekiki-To), kamishoyosan (Kami-shoyo-San), juzentaihoto (Juzen-taiho-To) and yokukansan (Yokukan-San) were selected as formulations compounding 1.5 g of Glycyrrhizae Radix. Lastly, chotosan (Choto-San), kamikihito (Kami-kihi-To), ninjinyoeito (Ninjin-yoei-To) and rikkunshito (Rikkunshi-To) were selected as formulations compounding 1.0 g of Glycyrrhizae Radix.

**Table 2:** List of Kampo prescriptions used in the study

shakuyakukanzoto (Shakuyaku-kanzo-To)	
Paeoniae Radix	4.0
Glycyrrhizae Radix	4.0
shoseiryuto (Sho-seiryu-To)	
Ephedra Herba	3.0
Paeoniae Radix	3.0
Zingiberis Processum Rhizoma	3.0
Glycyrrhizae Radix	3.0
Cinnamomi Cortex	3.0
Asiasari Radix	3.0
Schisandrae Fructus	3.0
Pinelliae Tuber	6.0
hangeshashinto (Hange-shashin-To)	
Pinelliae Tuber	5.0
Scutellariae Radix	2.5
Zingiberis Processum Rhizoma	2.5
Ginseng Radix	2.5
Glycyrrhizae Radix	2.5
Zizyphi Fructus	2.5
Copitidis Rhizoma	1.0
gorinsan (Gorin-San)	
Poria	5.0
Angelicae Radix	3.0
Scutellariae Radix	3.0
Glycyrrhizae Radix	2.0
Paeoniae Radix	2.0
Gardeniae Fructus	2.0
unkeito (Unkei-To)	
Pinelliae Tuber	5.0
Ophiopogonis Tuber	10.0
Angelicae Radix	2.0
Cnidii Rhizoma	2.0
Paeoniae Radix	2.0
Ginseng Radix	2.0
Cinnamomi Cortex	2.0
Moutan Cortex	2.0
Glycyrrhizae Radix	2.0
Zingiberis Rhizoma	0.3
Euodiae Fructus	3.0
Asini Corii Collas	2.0
bakumondoto (Bakumondo-To)	
Ophiopogonis Tuber	10.0
Pinelliae Tuber	5.0
Zizyphi Fructus	3.0
Ginseng Radix	2.0
Glycyrrhizae Radix	2.0
Oryzae Fructus	5.0
boiogito (Boi-ogi-To)	
Sinomeni Caulis et Rhizoma	4.0
Astragali Radix	5.0
Atractylodis Rhizoma	3.0
Zingiberis Rhizoma	1.0
Zizyphi Fructus	4.0
Glycyrrhizae Radix	2.0
bofutsushosan (Bofu-tsusho-San)	
Angelicae Radix	1.2
Paeoniae Radix	1.2
Cnidii Rhizoma	1.2
Gardeniae Fructus	1.2
Forsythiae Fructus	1.2
Menthae Herba	1.2
Zingiberis Rhizoma	0.4
Schizonepetae Spica	1.2
Saposhnikoviae Radix	1.2
Ephedra Herba	1.2
Natrium Sulfuricum	0.6
Atractylodis Rhizoma	2.0
Platycodi Radix	2.0
Scutellariae Radix	2.0
Glycyrrhizae Radix	2.0
Gypsum Fibrosum	2.0
Kasseki	3.0
saireito (Sai-rei-To)	
Bupleuri Radix	5.0
Pinelliae Tuber	4.0
Zingiberis Rhizoma	1.0
Scutellariae Radix	3.0
Zizyphi Fructus	2.5
Ginseng Radix	2.5
Glycyrrhizae Radix	2.0
Alismatis Tuber	5.0
Polyporus	3.0
Poria	3.0
Atractylodis Rhizoma	3.0
Cinnamomi Cortex	2.5
shosaikoto (Sho-saiko-To)	
Bupleuri Radix	6.0
Pinelliae Tuber	5.0
Scutellariae Radix	3.0
Ginseng Radix	3.0
Zizyphi Fructus	3.0
Zingiberis Rhizoma	1.0
Glycyrhizae Radix	2.0
saibokuto (Sai-boku-To)	
Bupleuri Radix	7.0
Pinelliae Tuber	5.0
Zingiberis Rhizoma	1.0
Scutellariae Radix	3.0
Zizyphi Fructus	3.0
Ginseng Radix	3.0
Glycyrrhizae Radix	2.0
Poria	5.0
Magnoliae Cortex	3.0
Perillae Herba	2.0
kakkonto (Kakkon-To)	
Puerariae Radix	8.0
Ephedra Herba	4.0
Zingiberis Rhizoma	1.0
Zizyphi Fructus	4.0
Cinnamomi Cortex	3.0
Paeoniae Radix	3.0
Glycyrrhizae Radix	2.0
keishikaryukotsuboreito (Keishi-ka-ryukotsu-borei-To)	
Cinnamomi Cortex	3.0
Paeoniae Radix	3.0
Zizyphi Fructus	3.0
Zingiberis Rhizoma	3.0
Glycyrrhizae Radix	2.0
Fossilia Ossis Mastodi	2.0
Ostreae Testa	3.0
maoto (Mao-To)	
Ephedra Herba	4.0
Armeniacae Semen	4.0
Cinnamomi Cortex	3.0
Glycyrrhizae Radix	1.5
saikokeishito (Saiko-keishi-To)	
Bupleuri Radix	5.0
Pinelliae Tuber	4.0
Cinnamomi Cortex	2.0
Paeoniae Radix	2.0
Scutellariae Radix	2.0
Ginseng Radix	2.0
Zizyphi Fructus	2.0
Glycyrhizae Radix	1.5
Zingiberis Rhizoma	1.0
seishinrenshiin (Seishin-renshi-In)	
Ophiopogonis Tuber	4.0
Poria	4.0
Ginseng Radix	3.0
Plantaginis Semen	3.0
Scutellariae Radix	3.0
Astragali Radix	2.0
Glycyrhizae Radix	1.5
Nelumbis Semen	4.0
Lycii Cortex	2.0
yokukansankachinpihange (Yokukan-San-ka-chinpi-hange)	
Angelicae Radix	3.0
Cnidii Rhizoma	3.0
Poria	4.0
Atractylodis Rhizoma	4.0
Bupleuri Radix	2.0
Pinelliae Tuber	5.0
Glycyrhizae Radix	1.5
Citri Unshu Pericarpium	3.0
Uncariae Uncis cum Ramulus	3.0
hochuekkito (Hochu-ekki-To)	
Ginseng Radix	4.0
Atractylodis Rhizoma	4.0
Astragali Radix	4.0
Angelicae Radix	3.0
Citri Unshu Pericarpium	2.0
Zizyphi Fructus	2.0
Bupleuri Radix	1.0
Glycyrhizae Radix	1.5
Zingiberis Rhizoma	0.5
Cimicifugae Rhizoma	0.5
kamishoyosan (Kami-shoyo-San)	
Angelicae Radix	3.0
Atractylodis Rhizoma	3.0
Bupleuri Radix	3.0
Gardeniae Fructus	2.0
Zingiberis Rhizoma	1.0
Paeoniae Radix	3.0
Poria	3.0
Moutan Cortex	2.0
Glycyrhizae Radix	1.5
Menthae Herba	1.0
juzentaihoto (Juzen-taiho-To)	
Ginseng Radix	3.0
Astragali Radix	3.0
Atractylodis Rhizoma	3.0
Poria	3.0
Angelicae Radix	3.0
Paeoniae Radix	3.0
Rehmanniae Radix	3.0
Cinnamomi Cortex	3.0
Glycyrhizae Radix	1.5
yokukansan (Yokukan-San)	
Angelicae Radix	3.0
Cnidii Rhizoma	3.0
Poria	4.0
Atractylodis Rhizoma	4.0
Bupleuri Radix	2.0
Glycyrhizae Radix	1.5
Uncariae Uncis cum Ramulus	3.0
chotosan (Choto-San)	
Uncariae Uncis cum Ramulus	3.0
Citri Unshu Pericarpium	3.0
Chrysanthemi Flos	2.0
Saposhnikoviae Radix	2.0
Pinelliae Tuber	3.0
Ophiopogonis Tuber	3.0
Poria	3.0
Ginseng Radix	2.0
Zingiberis Rhizoma	1.0
Glycyrhizae Radix	1.0
Gypsum Fibrosum	5.0
kamikihito (Kami-kihi-To)	
Ginseng Radix	3.0
Poria	3.0
Longan Arillus	3.0
Angelicae Radix	2.0
Bupleuri Radix	3.0
Glycyrhizae Radix	1.0
Zizyphi Fructus	2.0
Zingiberis Rhizoma	0.5
Atractylodis Rhizoma	3.0
Zizyphi Semen	3.0
Astragali Radix	3.0
Polygalae Radix	2.0
Gardeniae Fructus	2.0
Saussreae Raidx	1.0
Moutan Cortex	2.0
ninjinyoeito (Ninjin-yoei-To)	
Ginseng Radix	3.0
Angelicae Radix	4.0
Paeoniae Radix	2.0
Rehmanniae Radix	4.0
Atractylodis Rhizoma	4.0
Poria	4.0
Cinnamomi Cortex	2.5
Astragali Radix	1.5
Citri Unshu Pericarpium	2.0
Polygalae Radix	2.0
Schisandrae Fructus	1.0
Glycyrhizae Radix	1.0
rikkunshito (Rikkunshi-To)	
Ginseng Radix	4.0
Atractylodis Rhizoma	4.0
Poria	4.0
Pinelliae Tuber	4.0
Citri Unshu Pericarpium	2.0
Zizyphi Fructus	2.0
Glycyrhizae Radix	1.0
Zingiberis Rhizoma	0.5

**Fig. 2:**
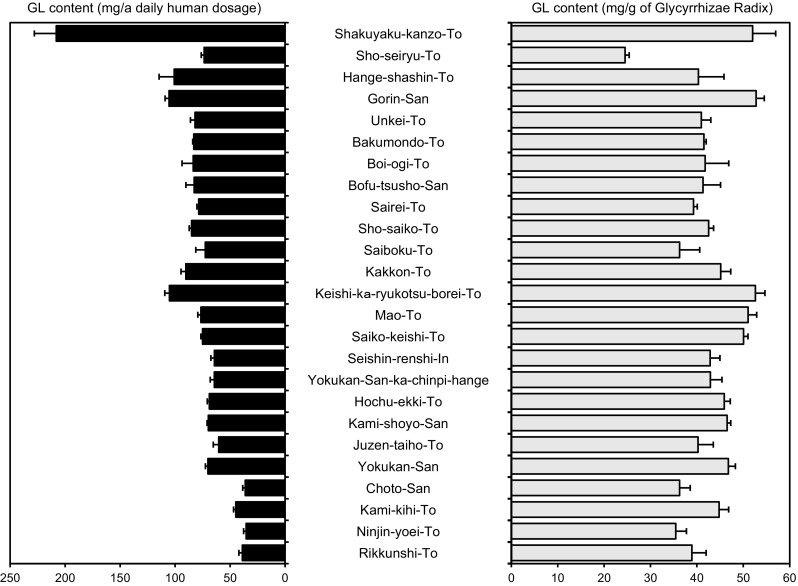
Determination of glycyrrhizin (GL) content in 25 kinds of Kampo extracts. Each *column* represents the mean ± SEM of three samples

**Fig. 3:**
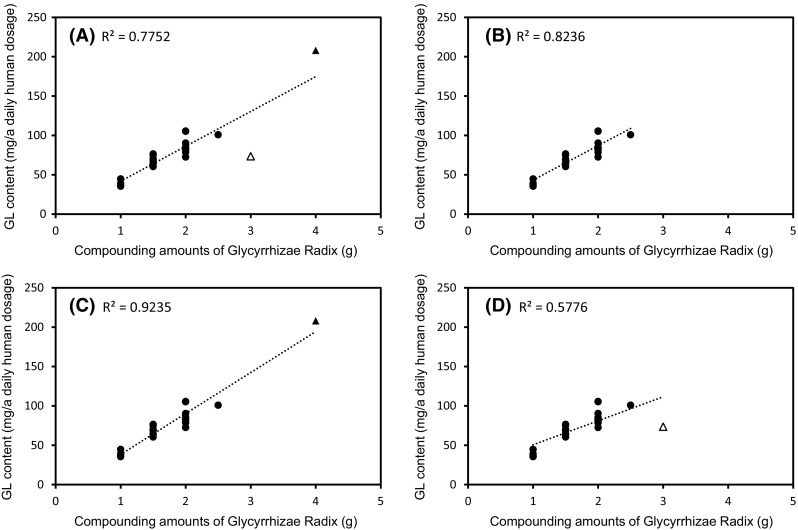
The relationship between glycyrrhizin (GL) content and amount of Glycyrrhizae Radix compounding 25 kinds of Kampo extracts. **a** 25 kinds of Kampo extracts, **b** 23 kinds of Kampo extracts excluding Sho-seiryu-To and Shakuyaku-kanzo-To, **c** 24 kinds of Kampo extracts excluding Sho-seiryu-To, **d** 24 kinds of Kampo extracts excluding Shakuyaku-kanzo-To. Each *point* represents the mean of three samples. *Filled circle* 23 kinds of Kampo extracts, *open triangle* Sho-seiryu-To, *filled triangle* Shakuyaku-kanzo-To

As shown in Fig. 2 (see also Supplement Table 1), the GL content per daily dosage in each Kampo medicine is generally proportional to the compounding amount of Glycyrrhizae Radix. Figure 3a shows the relationship between the GL content and the compounding amount of Glycyrrhizae Radix. The coefficient of determination among 25 formulas is 0.7752, and Sho-seiryu-To significantly deviates downward from the correlation curve while Shakuyaku-kanzo-To seems to be highly placed above this correlation curve. When we re-calculate the coefficient of determination among 23 or 24 formulas other than Sho-seiryu-To and/or Shakuyaku-kanzo-To, we can see the comparatively good linearity and higher value of the determination coefficient (*r* ^2^ = 0.9235) if we save and except Sho-seiryu-To (Fig. 3b–d). Thus, we can regard Sho-seiryu-To as an aberration to this observation.

### Effects of other crude drugs on GL content in Sho-seiryu-To

In our preliminary experiments, we prepared extracts by combining Glycyrrhizae Radix with other components compounding Sho-seiryu-To such as Pinelliae Tuber, Ephedra Herba, Cinnamomi Cortex, Schisandrae Fructus, Paeoniae Radix, Asiasari Radix, and Zingiberis Processum Rhizoma. Then, the GL content of every combination was determined by HPLC. We observed a significant decrease of GL content when Glycyrrhizae Radix was combined with Schisandrae Fructus containing Sho-seiryu-To (data not shown).

**Fig. 4:**
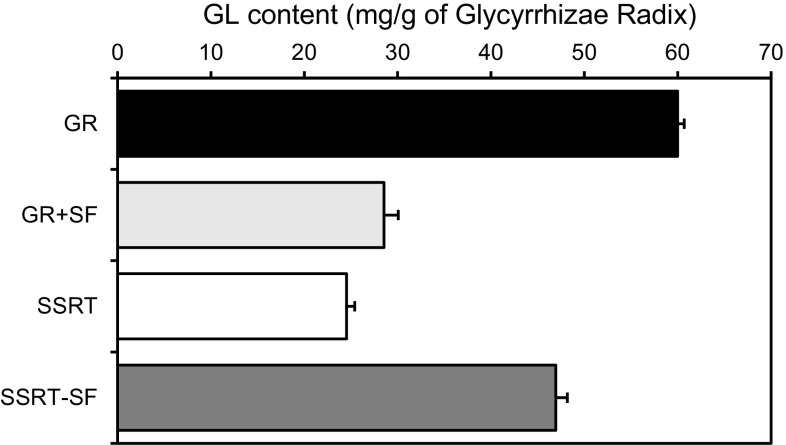
Effect of Schisandrae Fructus on the extraction efficiency of glycyrrhizin (GL) in Sho-seiryu-To. Each *column* represents the mean ± SEM of three samples

In order to confirm the effect of Schisandrae Fructus on GL content in Sho-seiryu-To, we prepared the extract of Glycyrrhizae Radix alone, the extract of Glycyrrhizae Radix combined with Schisandrae Fructus, and the extract of Sho-seiryu-to without Schisandrae Fructus, and determined the GL content. As shown in Fig. 4, the GL content of the extract with Schisandrae Fructus was about half of that in the extract of Glycyrrhizae Radix alone and nearly equal to that in Sho-seiryu-To. Furthermore, the GL content increased significantly when Sho-seiryu-To was prepared without Schisandrae Fructus. These results suggest that the low content of GL in Sho-seiryu-To is due to Schisandrae Fructus.

**Fig. 5:**
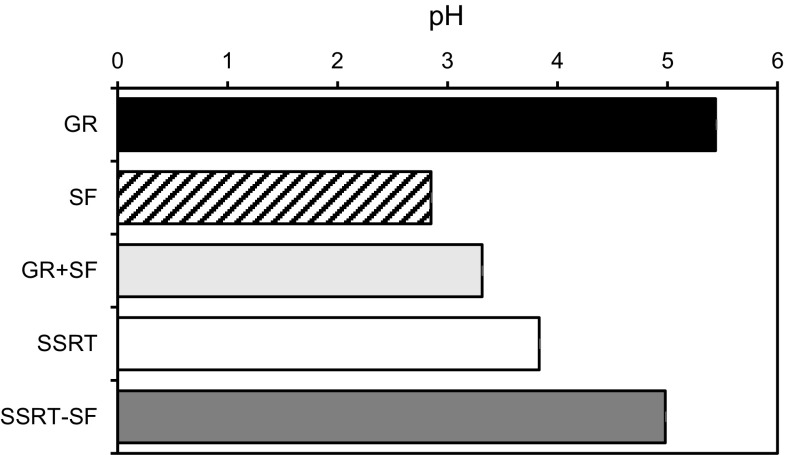
Effect of Schisandrae Fructus on the pH value of the decoction in Sho-seiryu-To. Each *column* represents the mean ± SEM of three samples

Schisandrae Fructus is the fruit of *Schisandra chinensis* (Schisandraceae) and contains lignans such as schizandrin and gomisin A. It has been reported that it also contains organic acids such as citric acid, malic acid and tartaric acid [25]. Due to the organic acids, the pH value could be lowered and the extraction efficiency of GL could be decreased. In connection with this, we also measured the pH value of the decoctions. As shown in Fig. 5, the pH value in the decoction with Schisandrae Fructus was lower and its pH was <3. The pH of the decoction of Glycyrrhizae Radix alone was 5.44, the decoction with Schisandrae Fructus was 3.31, and that of the decoction of Sho-seiryu-To was 3.83. The pH of the decoction of Sho-seiryu-to without Schisandrae Fructus increased to 4.98. These results are comparable to the results of the GL content in each extract; therefore, we think that the pH in the decoction is a critical factor that could affect the extraction efficiency of GL. Okamura et al. have already reported that low pH due to the organic acids of Schisandrae Fructus in the decoction caused the inhibition of GL dissolution in Sho-seiryu-To when they demonstrated simultaneous HPLC determination of puerarin, daizein, paeoniflorin, liquiritin, cinnamic acid cinnamaldehyde and GL in 8 kinds of Kampo formulas containing Ephedra Herba [26]. We observed that our findings in the case of Sho-seiryu-To are consistent with their results. On the other hand, Ninjin-yoei-to also contain Schisandrae Fructus but the GL content and the pH value of the decoction were not affected. We speculate that because the amount of Schisandrae Fructus in Ninjin-yoei-To is smaller resulting in less organic acid, the effect is not as profound as in Sho-seiryu-To (Fig. 2). Furthermore, we looked for other crude drugs containing organic acids like Schisandrae Fructus and we found Corni Fructus, fruits of *Cornus officinalis* (Cornaceae). Kampo formulas containing Corni Fructus are Gosha-jinki-Gan, Hachimi-jio-Gan and Rokumi-jio-Gan in ethical and OTC formulations, but fortunately these Kampo prescriptions do not contain Glycyrrhizae Radix. Therefore, the combination between Glycyrrhizae Radix and Corni Fructus was not investigated.

### Effect of pH on the extraction efficiency of GL from Glycyrrhizae Radix

We clarified the effect of pH on the extraction efficiency of GL from Glycyrrhizae Radix using powdered crude drug and various buffers.

**Fig. 6:**
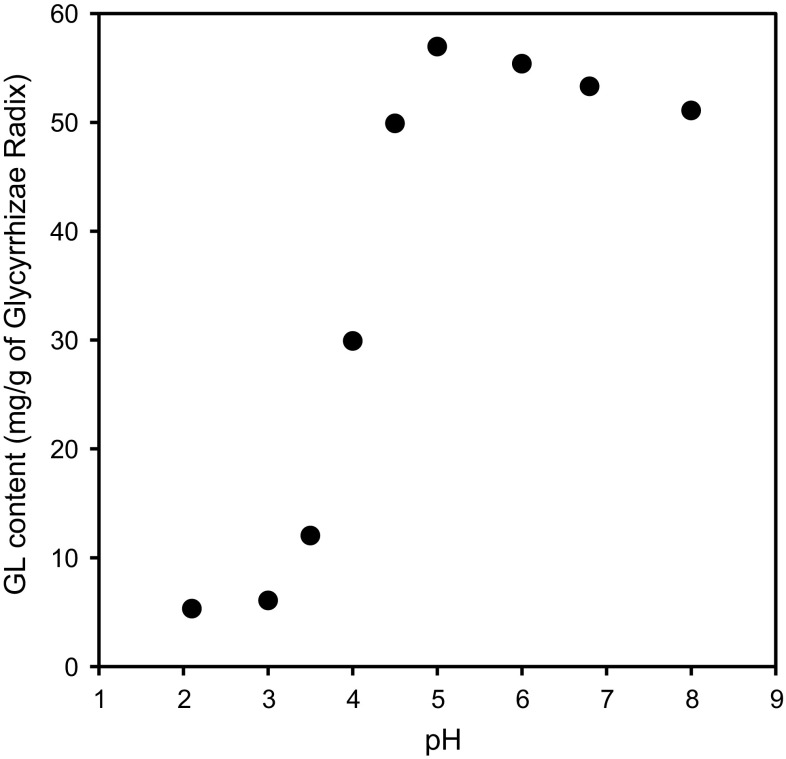
The relationship between the extraction efficiency of glycyrrhizin (GL) and the pH value of buffer. Each *point* represents the mean of three samples

As shown in Fig. 6, the extraction efficiency showed the sigmoid curve relative to pH. GL was not extracted in the buffer at pH 2.1 and pH 3. It was then gradually dissolved until the extraction efficiency reached maximum at pH 5.0, and a slight decrease in dissolution was observed with enhanced pH value. Since the pKa value of GL has been reported as pKa1 = 3.98, pKa2 = 4.62 and pKa3 = 5.17 [27], the dissolution behavior may be explained by the pKa value of GL.

**Fig. 7:**
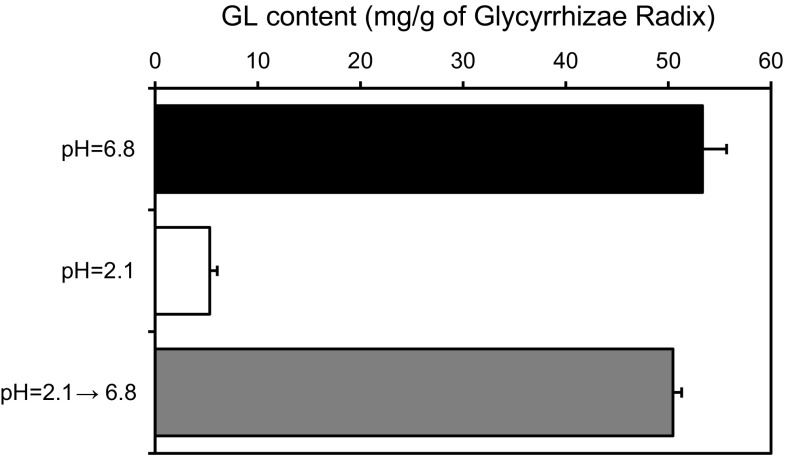
Effect of pH on the extraction efficiency of glycyrrhizin (GL) from Glycyrrhizae Radix: recovery of the extraction efficiency of GL extracted with 100 mM phosphate buffer (pH = 6.8) from the residue pre-extracted with 100 mM phosphate buffer (pH = 2.1). Each *column* represents the mean of three samples

In addition, we verified the effect of the pH value of the solvent on the extraction efficiency of GL from Glycyrrhizae Radix. The powdered Glycyrrhizae Radix was extracted with 100 mM phosphate buffer at pH 2.1 or pH 6.8. As expected, the GL content in the buffer at pH 2.1 was lower than in the buffer at pH 6.8. Next, the residue extracted with a buffer at pH 2.1 was re-extracted with a buffer at pH 6.8 and eventually GL thought to be contained in the residue was recovered quantitatively from the buffer with pH 6.8 (Fig. 7). Thus, we have confirmed that the extraction efficiency of GL from its crude drug is dependent on the pH value of the decoction.

**Fig. 8:**
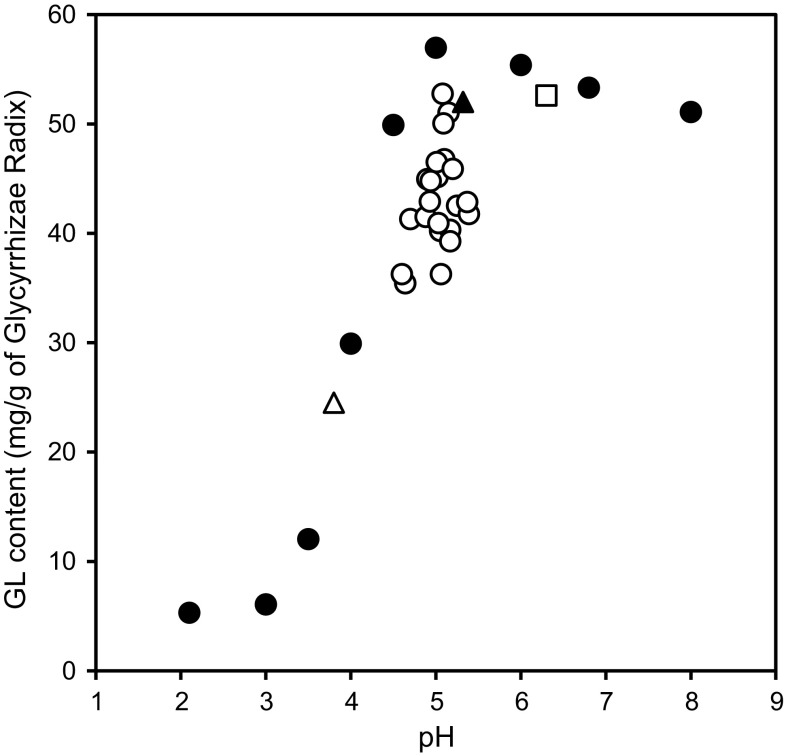
The relationship between the extraction efficiency of glycyrrhizin (GL) and the pH values of 25 kinds of Kampo extracts. Each *point* represents the mean of three samples. *Open circle* buffer, *filled circle* 22 kinds of Kampo extracts, *open triangle* Sho-seiryu-To, *filled traingle* Shakuyaku-kanzo-To, *open square* Keishi-ka-ryukotsu-borei-To

Lastly, we measured the pH value of the decoction of the 25 kinds of Kampo extracts used in the study and the data were added to the top of the graph in Fig. 6 (Fig. 8). Interestingly, Keishi-ka-ryukotsu-borei-To showed a relatively high pH value (pH 6.3) and its mean GL content was 52.6 mg/g of the crude drug. Keishi-ka-ryukotsu-borei-To contains Fossilia Ossis Mastodi and Ostea Testa, and it has been reported that the pH value can be around the neutral range because of the calcium carbonate present in the mentioned crude drugs [28]. The correlation analysis showed that the extraction efficiency correlates significantly with the pH value of the decoction (*r* = 0.7101, *p* < 0.0001). These results suggest that the extraction efficiency of GL is basically dependent on the pH value of the decoction, but other factors such as adsorption to other crude drugs compounding Kampo formulas could also decrease the GL content in some Kampo prescriptions [29–32].

### The relationship between the GL content and the incidence of adverse effects of Kampo medicine containing Glycyrrhizae Radix

**Fig. 9:**
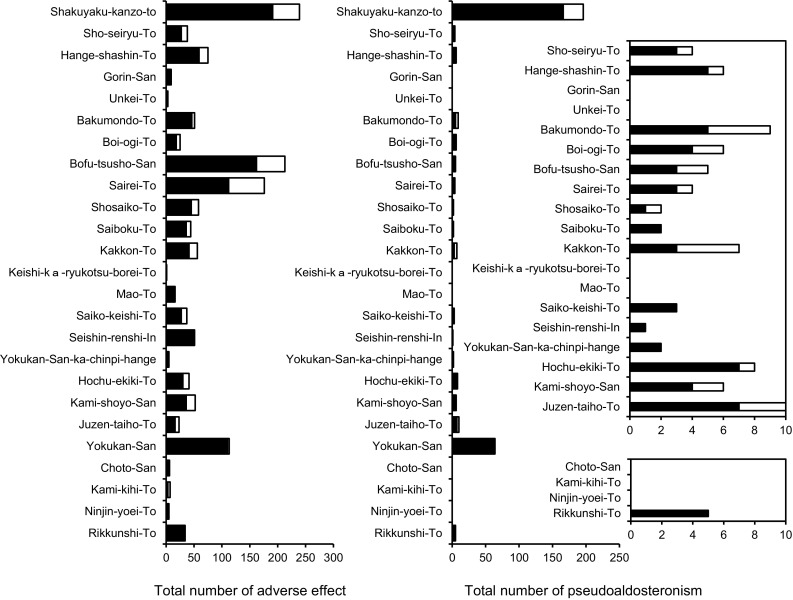
The incidence of adverse effects of Kampo formulas containing Glycyrrhizae Radix. *Closed column* represents the incidence from using ethical Kampo extract formulations and *opened column* represents the incidence from using OTC Kampo extract formulations, respectively

Since the GL content in a daily dosage of Sho-seiryu-To is comparatively lower than the other Kampo prescriptions used in the study, we became interested with the incidence of adverse effects of Sho-seiryu-To. The incidence of adverse effects of Kampo prescriptions used in the study was investigated using the Japanese Adverse Drug Event Report (JADER) database from 2004−2015 by the Pharmaceuticals and Medical Devices Agency (PMDA) in Japan [33]. We chose five preferred terms (pseudoaldosteronism, hypokalemia, hypokalemic syndrome, myopathy and rhabdomyolysis) for typical Glycyrrhizae Radix-induced adverse events in the investigation. As shown in Fig. 9, the most reported cases in Shakuyaku-kanzo-To were due to Glycyrrhizae Radix, and the same tendency was observed with Yokukan-San. In the case of Sho-seiryu-To, the low GL content could be the reason for the low incidence of pseudoaldosteronism in addition to the short-term use for nose allergy. On the other hand, Hochu-ekki-To and Juzen-taiho-To which contain a lower amount of GL have numerous reported cases related to Glycyrrhizae Radix. This observation may be due to their long-term administration for elderly patients. In addition, we evaluated the relationship between the compounding amounts of Glycyrrhizae Radix in 25 kinds of Kampo formulas and the number of reported cases concerning Glycyrrhizae Radix-induced adverse events and we found that the compounding amounts may be associated with pseudoaldosteronism (*r* = 0.6447, *p* < 0.0005). Moreover, we found much better correlation when the GL content was considered instead of the compounding amount of Glycyrrhizae Radix (*r* = 0.7757, *p* < 0.0001). These results suggest that the actual GL content is a better index to consider in order to avoid the adverse effects of Glycyrrhizae Radix-containing formulas. Since there are numerous variations of Glycyrrhizae Radix on the market, it is also possible that there is a certain level of variation in the GL content among the manufacturers of pharmaceuticals even in the same Kampo formula. Thus, further analytical and epidemiological studies are needed.

## Conclusion

In this study, we determined the GL content of the 25 major kinds of Kampo extracts compounding Glycyrrhizae Radix in Japan. We found that the GL content per daily dosage in each Kampo medicine is generally proportional to the compounding amount of Glycyrrhizae Radix. We also clarified that the extraction efficiency of GL in the decoction is not constant and is basically dependent on the pH value of the decoction. Moreover, the correlation analysis with Glycyrrhizae Radix-induced adverse events obtained from JADER suggested that the actual GL content is a better index to consider in order to avoid the adverse effects of Glycyrrhizae Radix-containing Kampo formulas.

## Electronic supplementary material

Supplementary material 1 (DOCX 23 kb)
