# Correction to: Comparison of glycyrrhizin content in 25 major kinds of Kampo extracts containing Glycyrrhizae Radix used clinically in Japan

**DOI:** 10.1007/s11418-018-1195-9

**Published:** 2018-03-01

**Authors:** Mitsuhiko Nose, Momoka Tada, Rika Kojima, Kumiko Nagata, Shinsuke Hisaka, Sayaka Masada, Masato Homma, Takashi Hakamatsuka

**Affiliations:** 1 Department of Pharmacognosy, Faculty of Pharmacy, Meijo University, 150 Yagotoyama, Tempaku-ku, Nagoya, Aichi 468-8503 Japan; 2 Division of Pharmacognosy, Phytochemistry and Narcotics, National Institute of Health Sciences, 1-18-1 Kamiyouga, Setagaya-ku, Tokyo, 158-8501 Japan; 3 Department of Pharmaceutical Sciences, Faculty of Medicine, University of Tsukuba, 1-1-1 Tenno-dai, Tsukuba, Ibaraki 305-8575 Japan

## Correction to: J Nat Med (2017) 71:711–722 10.1007/s11418-017-1101-x

The article Comparison of glycyrrhizin content in 25 major kinds of Kampo extracts containing Glycyrrhizae Radix used clinically in Japan, written by Mitsuhiko Nose, Momoka Tada, Rika Kojima, Kumiko Nagata, Shinsuke Hisaka, Sayaka Masada, Masato Homma and Takashi Hakamatsuka, was originally published Online First without open access. After publication in volume 71, issue 4, page 711–722 the author decided to opt for Open Choice and to make the article an open access publication. Therefore, the copyright of the article has been changed to © The Author(s) 2018 and the article is forthwith distributed under the terms of the Creative Commons Attribution 4.0 International License (http://creativecommons.org/licenses/by/4.0/), which permits use, duplication, adaptation, distribution and reproduction in any medium or format, as long as you give appropriate credit to the original author(s) and the source, provide a link to the Creative Commons license, and indicate if changes were made.

