# Supplementary material for: Comparison of glycyrrhizin content in 25 major kinds of Kampo extracts containing Glycyrrhizae Radix used clinically in Japan
Source: J Nat Med. 2017 Jun 12;71(4):711–22. doi: 10.1007/s11418-017-1101-x (PMC5897458; doi:10.1007/s11418-017-1101-x)
Supplement: Supplementary file 1 — Supplementary material 1 (DOCX 23 kb) [file 11418_2017_1101_MOESM1_ESM.docx]

Glycyrrhizin (GL) content

Kampo prescription Yield (g) mg/daily human dose mg/g of Glycyrrhizae Radix pH

shakuyakukanzoto (Shakuyaku-kanzo-To) 3.06 ± 0.07 208.03 ± 19.92 52.01 ± 4.98 5.32

shoseiryuto (Sho-seiryu-To) 5.46 ± 0.03 73.57 ± 2.66 24.52 ± 0.89 3.80

hangeshashinto (Hange-shashin-To) 6.27 ± 0.07 100.83 ± 13.74 40.33 ± 5.50 5.17

gorinsan (Gorin-San) 5.33 ± 0.02 105.55 ± 3.53 52.77 ± 3.06 5.08

unkeito (Unkei-To) 13.64 ± 0.09 81.90 ± 4.13 40.95 ± 2.06 5.03

bakumondoto (Bakumondo-To) 11.24 ± 0.08 83.03 ± 0.96 41.52 ± 0.48 4.88

boiogito (Boi-ogi-To) 7.13 ± 0.32 　　 83.53 ± 10.20 41.77 ± 5.10 5.39

bofutsushosan (Bofu-tsusho-San) 7.77 ± 0.18 82.63 ± 7.59 41.32 ± 3.79 4.70

saireito (Sai-rei-To) 9.00 ± 0.12 78.57 ± 1.61 39.28 ± 0.81 5.17

shosaikoto (Sho-saiko-To) 7.85 ± 0.11 85.05 ± 2.11 42.53 ± 1.06 5.25

saibokuto (Sai-boku-To) 8.28 ± 0.64 72.53 ± 8.67 36.27 ± 4.34 5.06

kakkonto (Kakkon-To) 7.81 ± 0.16 90.27 ± 4.33 45.13 ± 2.16 5.02

keishikaryukotsuboreito (Keishi-ka-ryukotsu-borei-To) 4.43 ± 0.08 105.20 ± 4.16 52.60 ± 2.08 6.30

maoto (Mao-To) 2.12 ± 0.03 76.55 ± 2.78 51.03 ± 1.86 5.15

saikokeishito (Saiko-keishi-To) 6.60 ± 0.05 75.09 ± 1.43 50.06 ± 0.95 5.09

seishinrenshiin (Seishin-renshi-In) 7.60 ± 0.10 64.30 ± 3.13 42.87 ± 2.09 5.37

yokukansankachinpihange (Yokukan-San-ka-chinpi-hange) 7.55 ± 0.11 64.36 ± 3.73 42.91 ± 2.49 4.93

hochuekkito (Hochu-ekki-To) 8.54 ± 0.25 68.86 ± 1.95 45.91 ± 1.30 5.20

kamishoyosan (Kami-shoyo-San) 6.94 ± 0.04 69.79 ± 1.16 46.52 ± 0.77 5.01

juzentaihoto (Juzen-taiho-To) 9.30 ± 0.05 60.36 ± 4.95 40.24 ± 3.30 5.05

yokukansan (Yokukan-San) 6.04 ± 0.01 70.17 ± 2.28 46.78 ± 1.52 5.10

chotosan (Choto-San) 6.35 ± 0.10 36.27 ± 2.29 36.27 ± 2.29 4.60

kamikihito (Kami-kihi-To) 10.90 ± 0.13 44.79 ± 2.06 44.79 ± 2.06 4.94

ninjinyoeito (Ninjin-yoei-To) 10.68 ± 0.01 35.45 ± 2.30 35.45 ± 2.30 4.64

rikkunshito (Rikkunshi-To) 6.16 ± 0.32 38.93 ± 3.06 38.93 ± 3.06 4.90
